# A181 EXPLORING THE ROLE OF *KLEBSIELLA VARIICOLA* AND *KLEBSIELLA PNEUMONIAE* IN PEDIATRIC ULCERATIVE COLITIS PATHOGENESIS

**DOI:** 10.1093/jcag/gwad061.181

**Published:** 2024-02-14

**Authors:** N Arjomand Fard, M Bording-Jorgensen, C Cheng, K Kerr, H Aujla, S Mansour, W Elhenawy, T Perry, E Wine

**Affiliations:** Physiology, University of Alberta, Edmonton, AB, Canada; Physiology, University of Alberta, Edmonton, AB, Canada; Physiology, University of Alberta, Edmonton, AB, Canada; Physiology, University of Alberta, Edmonton, AB, Canada; Physiology, University of Alberta, Edmonton, AB, Canada; Physiology, University of Alberta, Edmonton, AB, Canada; Physiology, University of Alberta, Edmonton, AB, Canada; Physiology, University of Alberta, Edmonton, AB, Canada; Physiology, University of Alberta, Edmonton, AB, Canada

## Abstract

**Background:**

Recent studies indicated that *Klebsiella (K) pneumoniae* (isolated from the stool of IBD patients) and *K. variicola* (isolated from the mesenteric tissue of Crohn disease patients) have the potential to induce inflammation in epithelial and preadipocyte cells, exacerbating colitis in murine models.

**Aims:**

We isolated these strains from pediatric ulcerative colitis (UC) patients' appendix (relevant to UC pathogenesis given the protective effects of appendectomy) and other non-inflamed colon sections. We hypothesized that these isolates are invasive and induce inflammation in UC. These strains have not been previously studied in UC.

**Methods:**

*K. variicola* and *K. pneumoniae* were isolated from two pediatric UC patients' appendices and other non-inflamed colon sections and identified using 16S DNA Sanger sequencing. Virulence of the two strains was determined by infecting Caco2 cell lines and performing adhesion and invasion assays, quantifying biofilm formation, and assessing barrier functions using transepithelial electrical resistance (TEER) measurement. Importantly, we monitored the production of pro-inflammatory (e.g., IL-8, TNF-α) and anti-inflammatory (IL-10) cytokines using reverse transcriptase quantitative polymerase chain reaction (RT-qPCR).

**Results:**

*K. variicola* and *K. pneumoniae* exhibited high levels of biofilm formation compared to adherent-invasive *Escherichia coli* (AIEC), which are commonly isolated from IBD patients. Furthermore, the *Klebsiella* strains adhered to epithelial cells within 2-3 hours post infection. TEER experiments showed compromised barrier integrity after 6 hours and overnight infection. Except for *K. pneumoniae* isolated from the ascending colon, the tested strains exhibited a significant increase in IL-8 expression. Similarly, *K. variicola* from the peri-appendicular region demonstrated elevated TNF-α gene expression.

**Conclusions:**

*K. variicola* and *K. pneumoniae* isolated from UC patients have the potential to form biofilms, disrupt barrier integrity, and trigger inflammatory responses. These findings unravel a potential role for pathogenic *Klebsiella* strains in driving UC pathogenesis. Importantly, these data shed light on the role of appendix-associated bacteria in the development of UC. Future work includes using comparative genomics to map the virulence determinants of these bacteria.

**Funding Agencies:**

CIHRWomen and Children's Health Research Institute (WCHRI)

